# Gene expression profiling of laterally spreading tumors

**DOI:** 10.1186/s12876-015-0295-1

**Published:** 2015-06-06

**Authors:** Shoko Minemura, Takeshi Tanaka, Makoto Arai, Kenichiro Okimoto, Arata Oyamada, Keiko Saito, Daisuke Maruoka, Tomoaki Matsumura, Tomoo Nakagawa, Tatsuro Katsuno, Takashi Kishimoto, Osamu Yokosuka

**Affiliations:** 1 Department of Gastroenterology and Nephrology, Graduate School of Medicine, Chiba University, 1-8-1 Inohana, Chiba, 260-8670 Japan; 2 Department of Molecular Pathology, Graduate School of Medicine, Chiba University, 1-8-1 Inohana, Chiba, 260-8670 Japan

**Keywords:** Laterally spreading tumor, Colon, Gene expression, Array analysis, BCL2L1

## Abstract

**Background:**

Laterally spreading tumors (LSTs) are generally defined as lesions >10 mm in diameter, are characterized by lateral expansion along the luminal wall with a low vertical axis. In contrast to other forms of tumor, LSTs are generally considered to have a superficial growth pattern and the potential for malignancy. We focused on this morphological character of LSTs, and analyzed the gene expression profile of LSTs.

**Methods:**

The expression of 168 genes in 41 colorectal tumor samples (17 LST-adenoma, 12 LST-carcinoma, 12 Ip [pedunculated type of the Paris classification)-adenoma, all of which were 10 mm or more in diameter] was analyzed by PCR array. Based on the results, we investigated the expression levels of genes up-regulated in LST-adenoma, compared to Ip-adenoma, by hierarchical and K-means clustering. To confirm the results of the array analysis, using an additional 60 samples (38 LST-adenoma, 22 Ip-adenoma), we determined the localization of the gene product by immunohistochemical staining.

**Result:**

The expression of 129 genes differed in colorectal tumors from normal mucosa by PCR array analysis. As a result of K-means clustering, the expression levels of five genes, *AKT1*, *BCL2L1*, *ERBB2*, *MTA2* and *TNFRSF25*, were found to be significantly up-regulated (*p* < 0.05) in LST-adenoma, compared to Ip-adenoma. Immunohistochemical analysis showed that the *BCL2L1* protein was significantly and meaningfully up-regulated in LST-adenoma compared to Ip-adenoma (*p* = 0.010). With respect to apoptosis status in LST-Adenoma, it assumes that *BCL2L1* is anti-apoptotic protein, the samples such as *BCL2L1* positive and TUNEL negative, or *BCL2L1* negative and TUNEL positive are consistent with the assumption. 63.2 % LST-adenoma samples were consistent with the assumption.

**Conclusions:**

LSTs have an unusual profile of gene expression compared to other tumors and *BCL2L1* might be concerned in the organization of LSTs.

**Electronic supplementary material:**

The online version of this article (doi:10.1186/s12876-015-0295-1) contains supplementary material, which is available to authorized users.

## Background

Colorectal tumors can be morphologically divided into three groups on the basis of their microscopic appearance: protruded-type, depressed-type and laterally spreading-type tumors [1, 2]. Although the protruded-type (polypoid) tumor has been the major focus of study, recently, the latter two types of tumor have received attention, not only from Japanese [3, 4] but also Westerners gastroenterologists [2, 5–7]. These two types of tumor have been reported to be the most difficult lesions to detect by endoscopic examination and to have the risk of being cancerous at the time of diagnosis. Although laterally spreading-type and depressed- type tumors progress in depth rather than spreading across the surface of the mucosa, the gene expression profile underlying the morphological pattern has not been determined.

Laterally spreading tumors (LSTs) are generally defined as lesions >10 mm in diameter, are characterized by lateral expansion along the luminal wall with a low vertical axis [3] and are usually divided into two types, granular-type (Gr-LST) and flat- or nongranular-type (NGr-LST), based on morphological differences determined endoscopically [7]. Gr-LST are composed of superficially spreading aggregates of nodules forming flat, broad-based lesions with a granulonodular and uneven surface, whereas NGr-LST are lesions with a flat, smooth surface without granulonodular features [2].

In this study, we aimed to determine the gene expression profile of LSTs, specifically, which genes are up or down regulated in comparison to polypoid tumors, and to identify the biological pathways involved, such as apoptosis, invasion, metastasis, cell cycling and signal transduction.

## Methods

### Sample collection and extraction of RNA

A total of 41 colorectal tumor samples resected by ESD (endoscopic submucosal dissection) or EMR (endoscopic mucosal dissection) from September 2011 to June 2013 at the Chiba University Hospital were selected for this study. The morphological classification and original pathological diagnoses were 17 LST-adenoma, 12 LST-carcinoma, and 12 Ip-adenoma, all 10 mm or more in diameter. Tumor and normal mucosa tissues were taken from the same patient during endoscopic treatment and incubated in RNA later® (Ambion, Inc., Austin, Texas USA) for 48 h at 4 °C, and then stored at −80 °C. Total RNA was extracted using TRIzol reagent (Invitrogen Co., Carlsbad, CA, USA) according to the manufacturer’s instructions and stored at −80 °C until further use. This study was approved by the ethics committee of Chiba University and written informed consent was obtained from all patients (UMIN 000009998).

### PCR array analysis

RNA samples were reversed transcribed using RT^2^ First Strand Kits (SA Biosciences Corp., Frederick, MD, USA). The resulting cDNA was analyzed using the Human Cancer Pathway and Human Extracellular Matrix & Adhesion Molecules RT^2^ Profiler™ PCR Arrays (SA Biosciences Corp.), which comprises a panel of 84 primer sets related to genes involved in the cytoskeleton, cell proliferation, cell-cell interactions and cell-matrix interactions. We examined 168 genes in total.

All results were normalized relative to expression of glyceraldehyde-3-phosphate dehydrogenase (*GAPDH)*. PCR arrays were analyzed using the 7300RT-PCR System (AB Applied Biosciences). The amplification protocol consisted of an initial denaturation at 95 °C for 10 min, followed by 40 cycles of denaturation at 95 °C for 15 s, and annealing and extension at 60 °C for 1 min. The expression of each gene was quantified based on its *C*t (cycle threshold), the number of cycles at which the linear phase crossed the threshold level.

### Gene expression data analysis

Statistical analysis of the PCR array data was performed using an online analysis tool (SA Biosciences Corp., http://pcrdataanalysis.sabiosciences.com/pcr/arrayanalysis.php), with statistical significance set at *p* ≤ 0.05. Relative changes in gene expression on quantitative RT-PCR were analyzed using the Δ*C*t or ΔΔ*C*t method. Data were normalized by subtracting the average *C*t value of the reference gene (GAPDH) from that of the sample genes in each group (17 LST-adenoma, 12 LST-carcinoma, 12 Ip-adenoma), with the normalized *C*t value designated the Δ*C*t value for that gene. Δ*C*t was compared to control samples using the equation ΔΔ*C*t = (Δ*C*t sample –Δ*C*t control) and fold changes in target gene expression were calculated using the formula, expression fold change = 2^(−ΔΔCt)^.

### Hierarchical and K-means clustering analysis

To demonstrate statistical significant difference in gene expression between LSTs and polypoid tumors, based on the results of PCR array analysis, we performed comparative statistical analyses, such as hierarchical and K-means clustering analysis, using GeneSpring GX 12.0 (Agilent) software (Chemical Evaluation and Research Institute, Saitama, Japan). Expression levels of 129 genes (the remaining 39 genes did not yield statistically efficient results in the PCR array analysis) in the 41 colorectal tumor samples were analyzed by hierarchical clustering. Based on these results, we performed K-means clustering to clarify the nature of gene expression in LSTs.

### Immunohistochemistry

To confirm the gene expression, immunohistochemical analysis was performed on 60 additional samples obtained from ESD or EMR for colorectal tumors from May 2003 to April 2013 at the Chiba University Hospital. These comprised 38 LST-adenoma and 22 Ip-adenoma samples. Sections of colorectal tumors resected by ESD or EMR were fixed and stained with hematoxylin-eosin for histological examination. We used the following antibodies: *TNFRSF25*, AKT1, *BCL2L1*, *MTA2* and *ERBB2* (1:200, Abcam plc., Cambridge, UK). Positively stained cells were visualized using 3’3-diaminobenzidine and counterstained with Mayer’s hematoxylin. Immunoreactivity was independently evaluated by two researchers who were blinded to patient outcome and pathology. We took the brown-colored cytoplasm cells as positive expressions. When more than half of cells showed the immunoreactive stains in one high power field, we judged as “positive” in this field. Then we selected nine high power fields randomly and judged each field respectively. If “positive” in more than half of fields was detected, we ultimately judged as “positive”. When there was any discrepancy in their evaluations, the samples were reexamined by both researchers.

### Evaluation of Apoptosis status in LST-Adenoma

To evaluate the apoptosis status in LST-adenoma, 38 LST-adenoma samples were analyzed by the terminal deoxynucleotidyl transferase (TdT)-mediated biotinylated deoxyuridine-triphosphate nick-end labeling (TUNEL) method. Apoptotic cell were identified by *In situ* Apoptosis Detection Kit (Takara Bio Inc., Shiga, Japan). TUNEL positivity was indicated by homogeneously brown nuclear staining. The apoptotic index was defined as the number of TUNEL-positive cells in randomly selected nine high power fields. When the apoptotic index was more than nine, we ultimately judged as “positive”.

### Statistical analysis

All results are expressed as means ± SD. Statistical differences between two groups were determined by Student’s *t*-test using SPSS 16.0 J (SPSS Inc., Chicago,IL, USA), with p < 0.05 considered statistically significant.

## Results

### Clinical characteristics of tumor samples

The 29 LSTs samples were from 15 males (51.7 %) and 14 females (48.3 %), of mean age 69.3 ± 11.8 years, and the mean tumor size was 23.1 ± 12.8 mm. In terms of location of LST, 16 (55.2 %) were located in the proximal portion (3 in cecum, 8 in ascending colon, 5 in transverse colon) and 13 (44.8 %) were located in the distal portion (2 in the descending colon, 4 in the sigmoid colon, 7 in the rectum). According to histological classification, 17 samples were adenoma and 12 were adenocarcinoma. The 12 polypoid tumor samples were from 10 males (83.3 %) and 2 females (16.7 %), of mean age 64.0 ± 13.1 years, and the mean tumor size was 16.3 ± 9.0 mm. Five (41.7 %) were located in the proximal portion (1 in the cecum, 4 in the ascending colon) and 7 (58.3 %) were located in the distal portion (1 in the descending colon, 4 in the sigmoid colon, 2 in the rectum) (Table 1).

**Table 1:** Baseline clinical characteristics of the patients whose tumors were used for PCR array analysis

Characteristics	LST	Ip
No. of samples	29	12
Age (years) (mean ± SD)	69.3 ± 11.8	64.0 ± 13.1
Sex (Male/Female)	15/14	10/2
Localization of tumor		
Cecum	3	1
Ascending	8	4
Transverse	5	0
Descending	2	1
Sigmoid	4	4
Rectum	7	2
Tumor size (mm) (mean ± SD)	23.1 ± 12.8	16.3 ± 8.9
Histological type		
Adenoma/Carcinoma	17/12	12/0

### Gene expression analysis by PCR array

The 129 genes which were expressed in colorectal tumors at a significantly different level (unpaired t-test, *p* < 0.05) to that of normal mucosa**,** and the ratios of their expression levels compared to normal mucosa (mean ± SD), are listed in the supplementary table (Additional file 1: Table S1).

### Hierarchical and K-means clustering

**Fig. 1:**
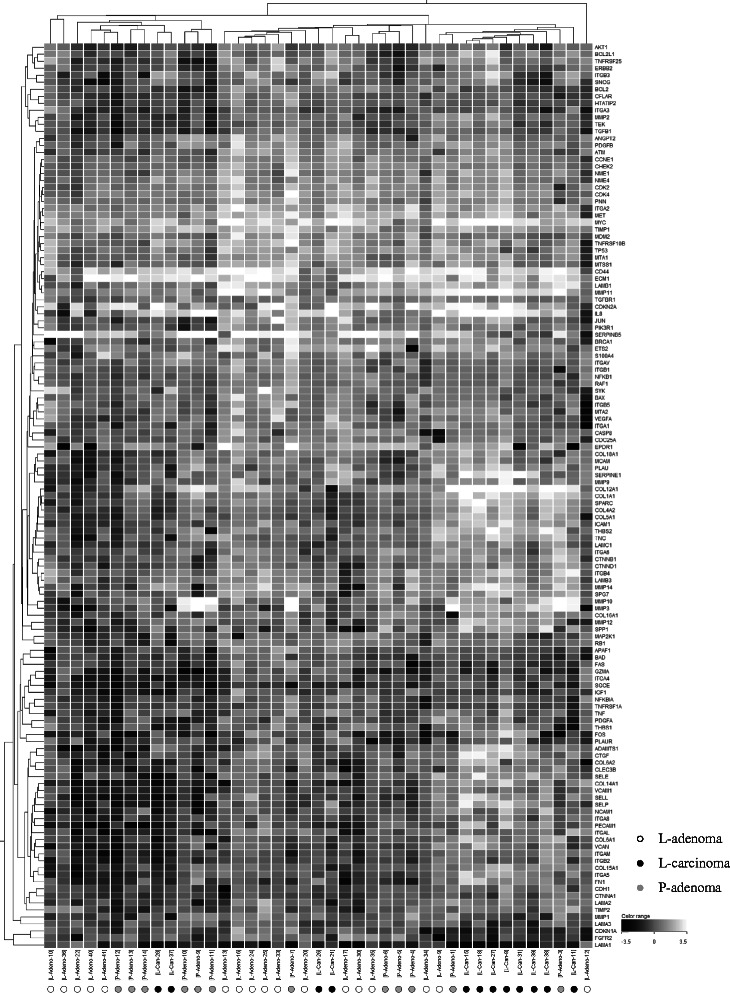
Hierarchical Clustering of Expression Levels in Colon Tumors. Expression levels of 129 genes in 41 colorectal tumor samples were analyzed by hierarchical clustering (distance metric: Peason Uncentered, linkage rule; Average). L-adenoma, LST-adenoma; L-carcinoma, LST-carcinoma; P-adenoma, Ip-adenoma. These samples were divided into 3 clusters. Group 1 consists of LST-adenomas and Ip-adenomas, Group 2 mainly consists of LST-adenomas and Group 3 mainly consists of LST-carcinomas

**Fig. 2:**
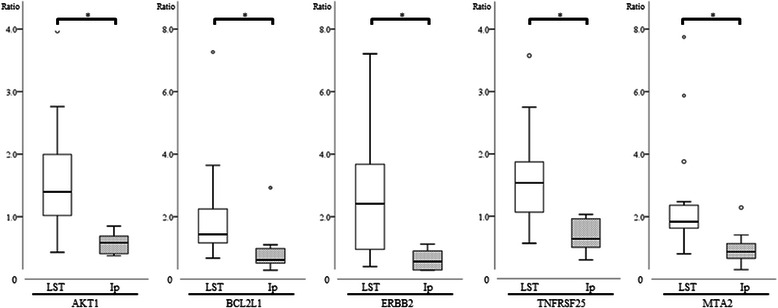
The mRNA Expression Level in Colon Adenoma. The ratios of mRNA expression level in adenomas compared to normal mucosa corrected by glyceraldehyde 3-phosphate dehydrogenase *(GAPDH)* were shown. White bars indicated LST-adenoma and dotted bars indicated Ip-adenoma. *p < 0.01, unpaired t-test. *AKT1*, v-akt murine thymoma viral oncogene homolog 1; *BCL2L1*, B cell leukemia/lymphoma 2-like1; *ERBB2*, v-erb-b2 avian erythroblastic leukemia viral oncogene homolog 2; *MTA2*, metastasis associated 1 family, member 2; *TNFRSF25*, tumor necrosis factor receptor superfamily, member 25

We performed hierarchical clustering using the gene expression data produced by PCR array analysis. The 41 colorectal tumor samples were divided into three large groups. Group 1 mainly consists of LST-adenoma and Ip-adenoma. Group 2 mainly consists of LST-adenoma. Group 3 mainly consists of LST-carcinoma (Fig. 1). By K-means clustering, the expression levels of five genes, *AKT1*, *BCL2L1*, ERBB2, *MTA2* and *TNFRSF25*, were seen to be significantly up-regulated (*p* <0.05) in LST-adenoma compared to Ip-adenoma (Fig. 2).

### Immunohistochemical staining

**Fig. 3:**
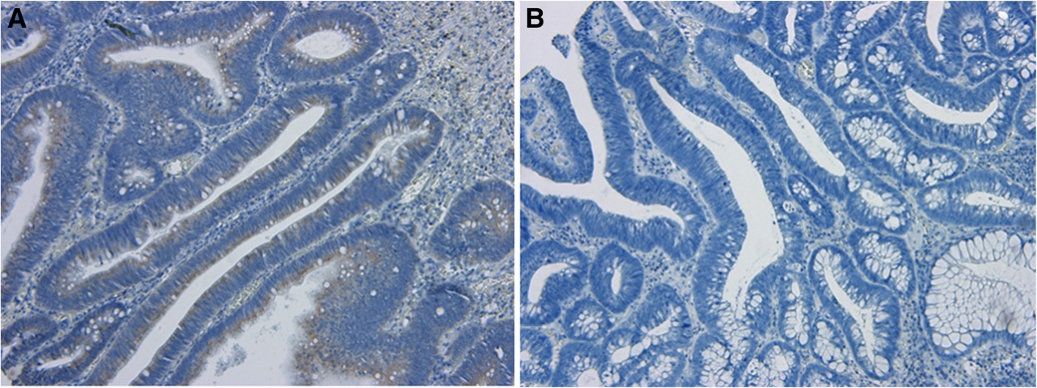
Immunohistochemical analysis of BCL2L1 expression in LST- and Ip-adenoma. **a** Positive expression in LST-adenoma (×100). **b** Negative expression in Ip-adenoma (×100)

Immunohistochemical staining showed that *BCL2L1* and *TNFRSF25* proteins were significantly up-regulated in LST-adenoma, compared to Ip-adenoma (*p* = 0.010, *p* < 0.001). Of the LST-adenomas, 25 (66 %) were *BCL2L1* positive and 38 (100 %) were *TNFRSF25* positive. On the other hand, of the IP-adenomas, 7 (32 %) were *BCL2L1* positive and 10 (45 %) were *TNFRSF25* positive (Table 2). *BCL2L1* protein expression was localized in the cytoplasm of the tumor cells (Fig. 3). There was no difference between LST-adenoma and Ip-adenoma in expression of the other proteins, *AKT1*, *MTA2*, and *ERBB2*. We did not get meaningful immunohistochemical staining patterns for the *AKT*1 and *ERBB2* proteins but the *MTA2* protein was found to be up-regulated in both LST-Adenoma and Ip-Adenoma compared to normal mucosa. In high grade adenoma, 14 out of 20 LST cases (70 %) showed high expression of *BCL2L1* by IHC, on the contrary, only 7 out of 17 Ip adenoma cases (41 %) showed high expression of it (*p* = 0.078, chi-squared test).

**Table 2:** Immunohistochemical staining of BCL2L1 in LST- and Ip-adenoma

IHC staining	Adenoma		*P*-value
	LST positive/negative	Ip positive/negative	
TNFRSF25	38/0	10/12	<0.001
AKT1	5/33	4/18	0.414
BCL2L1	25/13	7/15	0.010
MTA2	38/0	22/0	-
ERBB2	2/36	0/22	0.274

### Apoptosis status in LST-Adenoma

In 38 LST-adenoma, 7 (18.4 %) sample was TUNEL positive. It assumes that *BCL2L1* is anti-apoptotic protein, the samples such as *BCL2L1* positive and TUNEL negative, or *BCL2L1* negative and TUNEL positive are consistent with the assumption. We pathologically classified into high-grade dysplasia and low-grade dysplasia of 38 LST-adenoma. In 18 high-grade LST-adenoma, ten were *BCL2L1* positive and TUNEL negative and two were *BCL2L1* negative and TUNEL positive. In 20 low-grade LST-adenoma, ten were *BCL2L1* positive and TUNEL negative, two were *BCL2L1* negative and TUNEL positive. In summary, 22(63.2 %) samples were consistent with the assumption.

## Discussion

The morphological features of tumors are closely related to their growth patterns [8]. Polypoid tumors are believed to exhibit a predominantly vertical growth pattern, rather than a horizontal growth pattern, while non-polypoid tumors are believed to exhibit the opposite pattern, resulting in horizontal growth. Although there are some reports that LSTs have distinct biological characteristics compared to polypoid tumors [9, 10], the mechanism by which the LST conformation is generated remains unknown.

LSTs are believed to have distinct characteristics in terms of histological and genetic features [11]. Several molecular characteristics of LSTs have been described, including alteration of the adenomatous polyposis coli (APC) gene or β-catenin [12–14] affecting the WNT/APC/β-catenin signaling pathway, mutation of v-Ki-ras2 Kirsten rat sarcoma viral oncogene homolog (KRAS) [12, 15–20]. However, the molecular background of LSTs has remained largely unknown [19, 21]. Most of these studies have targeted genetic and/or epigenetic changes, or analyzed limited sets of genes. Our study is the first to clarify the gene expression profile by array analysis which could clarify the expression of many genes all at once. In addition, to avoid the effect of size, we used Ip type tumors with a diameter of 10 mm or more.

We performed immunohistochemical analysis of up-regulated genes which were show by PCR array on additional LSTs and polypoid tumors, the expression levels of *BCL2L1* in the LSTs were significantly higher than the polypoid tumors. This gene belongs to the Bcl-2 family whose members mainly comprise anti- or pro-apoptotic regulators that are involved in a wide variety of cellular activities [22, 23]. *BCL2L1* proteins are involved in the regulation of the mitochondrial pathway of apoptosis by controlling the production of reactive oxygen species and release of cytochrome c from mitochondria, both of which are potent inducers of apoptosis.

The growth of tumors represents an imbalance between cell proliferation and cell death [24]. There are some reports of analysis of cell proliferation markers, such as Ki-67 [25], apoptosis [26] and apoptosis related proteins, in colorectal tumors [27]. Kikuchi et al. also reported that the proliferative activity increased in adenomas with high-grade dysplasia and carcinomas among colorectal tumors, while apoptotic activity correspondingly increased in adenomas but significantly decreased in carcinomas [28]. Ki-67 is one of the reliable markers of cell cycle in adenoma or cancer cells. And apoptosis also involved in clinical or morphological characteristics of tumor. In fact, in a previous study, the expression levels of Ki-67 and Bcl-2 antigen in adenomatous colorectal polyps showed a good correlation [29]. Together with our results and other markers, it might lead to further understanding the pathophysiology of LST.

It is difficult to claim the significant difference of gene expressions determined the morphological features of LSTs. We analyzed the apoptosis status in LST-adenomas because *BCL2L1* worked as anti-apoptosis and the apoptosis status in adenoma was less complex than cancer. As a result, *BCL2L1* expression was related to apoptosis status in LST-adenoma samples. Our study based on PCR Array analysis and IHCs was merely not so much the analysis of gene function as the expression levels of mRNA and protein. Therefore, further study to clarify the relation between the gene function and clinical or morphological characteristics of LST has been needed. Similarly to us, Suzuki et al. reported that differences in both expression of anti-apoptotic gene Bcl-2 expression and apoptosis may play an important role in the morphogenesis of colorectal neoplasia [30]. Yamada et al. in non-polypoid colorectal adenomas (non-PAs), significant differences between apoptosis in the upper and lower portions of lesions suggests that vertical growth in non-PAs seems to be inhibited by apoptosis. Consequently, horizontal growth comes to dominate over vertical growth, resulting in the laterally spreading morphology of non-PAs [31]. Based on these reports, we suggest that anti-apoptotic gene Bcl-2 which have differential gene expression between LSTs-adenoma and Ip-adenoma may play an important role in the morphogenesis of LSTs by being responsible for apoptotic expression. Our analysis for apoptosis and gene expression has some limitations. We did not clarify the relation between *BCL2L1* and apoptosis directly in LST, and did not perform other methods except TUNNEL assay. And we could not show the question which the inverse correlation between *BCL2L1* and apoptosis in LST is occurred also in other types of tumor. Further analysis is needed. In the hierarchical clustering, cancer cases were not separated from adenoma ones perfectly. We suggested that a small number of genes analyzed by PCR array might cause this contingent result.

According to our IHC analysis, *TNFRSF25* protein was also up-regulated in LST-adenoma, compared to Ip-adenoma (*p* < 0.001). However, of the Ip-adenomas, 10 (45 %) were *TNFRSF25* positive. Up-regulation of *TNFRSF25* is not specific in LST-adenomas. Therefore, we speculated that the importance of *TNFRSF25* expression was less than that of *BCL2L1*.

## Conclusions

High expression level of apoptosis related genes, such as BCL2L1, might act as a trigger that determines tumor morphogenesis and be one of the genes responsible for horizontal growth, resulting in the laterally spreading morphology of LSTs.

## Additional file

Additional file 1: Table S1. Gene Lists of Expression Analysis by PCR array.

## Abbreviations

LSTs: Laterally spreading tumors
TUNEL: Terminal deoxynucleotidyl transferase (TdT)-mediated biotinylated deoxyuridine-triphosphate nick-end labeling
AKT1: v-akt murine thymoma viral oncogene homolog 1
BCL2L1: B cell leukemia/lymphoma 2-like1
ERBB2: v-erb-b2 avian erythroblastic leukemia viral oncogene homolog 2
MTA2: Metastasis associated 1 family, member 2
TNFRSF25: Tumor necrosis factor receptor superfamily, member 25
non-PAs: Non-polypoid colorectal adenomas
