# Supplementary material for: Gene expression profiling of laterally spreading tumors
Source: BMC Gastroenterol. 2015 Jun 6;15:64. doi: 10.1186/s12876-015-0295-1 (PMC4456718; doi:10.1186/s12876-015-0295-1)
Supplement: Additional file 1: Table S1. — Gene Lists of Expression Analysis by PCR array. [file 12876_2015_295_MOESM1_ESM.doc]

| **Table S1. Gene Lists of Expression Analysis by PCR array** | | | | | | | | | | | | |
| --- | --- | --- | --- | --- | --- | --- | --- | --- | --- | --- | --- | --- |
|  |  |  |  |  |  |  |  |  |  |  |  |  |
| Gene name | Description | LST-adenoma | | |  | LST-carcinoma | | |  | Ip-adenoma | | |
|  |  | (Fold change) | | |  | (Fold change) | | |  | (Fold change) | | |
| AKT1 | V-akt murine thymoma viral oncogene homolog 1 | 1.6 | ± | 0.8 |  | 1.2 | ± | 0.6 |  | 0.8 | ± | 0.5 |
| ANGPT2 | Angiopoietin 2 | 2.5 | ± | 1.9 |  | 2.0 | ± | 0.7 |  | 2.0 | ± | 1.3 |
| APAF1 | Apoptotic peptidase activating factor 1 | 1.4 | ± | 1.0 |  | 1.2 | ± | 0.5 |  | 1.2 | ± | 1.2 |
| ATM | Ataxia telangiectasia mutated | 2.4 | ± | 1.7 |  | 2.2 | ± | 1.2 |  | 1.7 | ± | 1.4 |
| BAD | BCL2-associated agonist of cell death | 1.1 | ± | 0.6 |  | 0.9 | ± | 0.3 |  | 0.6 | ± | 0.3 |
| BAX | BCL2-associated X protein | 2.2 | ± | 1.9 |  | 1.6 | ± | 0.6 |  | 1.6 | ± | 0.8 |
| BCL2 | B-cell CLL/lymphoma 2 | 2.0 | ± | 1.1 |  | 1.1 | ± | 0.5 |  | 1.2 | ± | 0.8 |
| BCL2L1 | BCL2-like 1 | 1.7 | ± | 0.7 |  | 3.3 | ± | 2.6 |  | 0.9 | ± | 0.6 |
| BRCA1 | Breast cancer 1, early onset | 1.9 | ± | 1.1 |  | 2.4 | ± | 1.1 |  | 2.2 | ± | 2.3 |
| CASP8 | Caspase 8, apoptosis-related cysteine peptidase | 2.2 | ± | 1.9 |  | 1.8 | ± | 0.4 |  | 1.6 | ± | 0.7 |
| CCNE1 | Cyclin E1 | 2.2 | ± | 1.0 |  | 2.5 | ± | 0.9 |  | 1.9 | ± | 1.3 |
| CDC25A | Cell division cycle 25 homolog A (S. pombe) | 1.7 | ± | 0.9 |  | 1.9 | ± | 0.8 |  | 1.7 | ± | 0.9 |
| CDK2 | Cyclin-dependent kinase 2 | 2.7 | ± | 1.5 |  | 2.6 | ± | 0.9 |  | 2.2 | ± | 1.3 |
| CDK4 | Cyclin-dependent kinase 4 | 3.3 | ± | 2.1 |  | 3.2 | ± | 1.5 |  | 3.0 | ± | 2.7 |
| CDKN1A | Cyclin-dependent kinase inhibitor 1A (p21, Cip1) | 1.2 | ± | 1.1 |  | 0.7 | ± | 0.3 |  | 0.9 | ± | 0.8 |
| CDKN2A | Cyclin-dependent kinase inhibitor 2A (melanoma, p16, inhibits CDK4) | 5.3 | ± | 4.5 |  | 9.6 | ± | 9.1 |  | 5.7 | ± | 3.9 |
| CFLAR | CASP8 and FADD-like apoptosis regulator | 1.3 | ± | 0.8 |  | 1.2 | ± | 0.4 |  | 1.0 | ± | 1.0 |
| CHEK2 | CHK2 checkpoint homolog (S. pombe) | 2.4 | ± | 1.3 |  | 2.1 | ± | 0.8 |  | 2.5 | ± | 1.7 |
| COL18A1 | Collagen, type XVIII, alpha 1 | 1.6 | ± | 0.9 |  | 2.4 | ± | 1.6 |  | 1.3 | ± | 0.9 |
| ERBB2 | V-erb-b2 erythroblastic leukemia viral oncogene homolog 2, | 2.0 | ± | 1.5 |  | 1.7 | ± | 0.8 |  | 1.0 | ± | 0.8 |
|  | neuro/glioblastoma derived oncogene homolog (avian) |  |  |  |  |  |  |  |  |  |  |  |
| ETS2 | V-Ets erythroblastosis virus E26 oncogene homolog 2 (avian) | 4.5 | ± | 4.1 |  | 3.0 | ± | 1.8 |  | 3.0 | ± | 2.6 |
| FAS | Fas (TNF receptor superfamily, member 6) | 1.3 | ± | 0.8 |  | 0.8 | ± | 0.4 |  | 1.0 | ± | 0.9 |
| FGFR2 | Fibroblast growth factor receptor 2 | 1.6 | ± | 1.2 |  | 0.9 | ± | 0.6 |  | 1.1 | ± | 0.8 |
| FOS | FBJ murine osteosarcoma viral oncogene homolog | 0.9 | ± | 0.8 |  | 2.5 | ± | 2.2 |  | 0.4 | ± | 0.4 |
| GZMA | Granzyme A (granzyme 1, cytotoxic T-lymphocyte-associated serine esterase 3) | 1.0 | ± | 0.7 |  | 0.8 | ± | 0.6 |  | 0.6 | ± | 0.7 |
| HTATIP2 | HIV-1 Tat interactive protein 2, 30 kDa | 1.4 | ± | 0.6 |  | 1.1 | ± | 0.3 |  | 1.3 | ± | 1.1 |
| IGF1 | Insulin-like growth factor 1 (somatomedin C) | 1.2 | ± | 1.0 |  | 0.7 | ± | 0.2 |  | 0.8 | ± | 0.6 |
| IL8 | Interleukin 8 | 4.3 | ± | 3.9 |  | 13.4 | ± | 12.1 |  | 6.2 | ± | 5.7 |
| ITGA1 | Integrin, alpha 1 | 2.0 | ± | 1.5 |  | 1.7 | ± | 1.1 |  | 1.5 | ± | 0.8 |
| ITGA2 | Integrin, alpha 2 (CD49B, alpha 2 subunit of VLA-2 receptor) | 4.3 | ± | 3.1 |  | 3.5 | ± | 1.6 |  | 3.5 | ± | 2.0 |
| ITGA3 | Integrin, alpha 3 (antigen CD49C, alpha 3 subunit of VLA-3 receptor) | 1.2 | ± | 0.9 |  | 1.2 | ± | 0.7 |  | 0.8 | ± | 0.5 |
| ITGA4 | Integrin, alpha 4 (antigen CD49D, alpha 4 subunit of VLA-4 receptor) | 1.1 | ± | 0.7 |  | 0.9 | ± | 0.5 |  | 0.7 | ± | 0.4 |
| ITGAV | Integrin, alpha V (vitronectin receptor, alpha polypeptide, antigen CD51) | 2.0 | ± | 1.2 |  | 1.7 | ± | 0.7 |  | 2.1 | ± | 1.2 |
| ITGB1 | Integrin, beta 1 (fibronectin receptor, beta polypeptide, antigen CD29 includes MDF2, MSK12) | 2.0 | ± | 1.1 |  | 1.5 | ± | 0.4 |  | 1.5 | ± | 1.1 |
| ITGB3 | Integrin, beta 3 (platelet glycoprotein IIIa, antigen CD61) | 1.6 | ± | 1.2 |  | 3.6 | ± | 2.5 |  | 1.2 | ± | 0.8 |
| ITGB5 | Integrin, beta 5 | 2.1 | ± | 1.2 |  | 1.8 | ± | 0.7 |  | 1.7 | ± | 1.6 |
| JUN | Jun proto-oncogene | 3.0 | ± | 3.0 |  | 2.4 | ± | 1.6 |  | 1.6 | ± | 1.5 |
| MAP2K1 | Mitogen-activated protein kinase kinase 1 | 1.5 | ± | 0.9 |  | 1.2 | ± | 0.7 |  | 1.3 | ± | 0.6 |
| MCAM | Melanoma cell adhesion molecule | 1.8 | ± | 1.1 |  | 2.0 | ± | 1.4 |  | 1.5 | ± | 1.0 |
| MDM2 | Mdm2 p53 binding protein homolog (mouse) | 2.7 | ± | 2.2 |  | 1.9 | ± | 0.9 |  | 2.4 | ± | 2.3 |
| MET | Met proto-oncogene (hepatocyte growth factor receptor) | 4.0 | ± | 1.9 |  | 3.6 | ± | 1.6 |  | 3.9 | ± | 2.7 |
| MMP1 | Matrix metallopeptidase 1 (interstitial collagenase) | 1.2 | ± | 0.7 |  | 0.9 | ± | 0.5 |  | 2.1 | ± | 1.2 |
| MMP2 | Matrix metallopeptidase 2 (gelatinase A, 72 kDa gelatinase, 72 kDa type IV collagenase) | 1.5 | ± | 0.9 |  | 2.0 | ± | 1.6 |  | 1.2 | ± | 0.8 |
| MMP9 | Matrix metallopeptidase 9 (gelatinase B, 92 kDa gelatinase, 92 kDa type IV collagenase) | 1.8 | ± | 1.8 |  | 2.9 | ± | 2.5 |  | 1.5 | ± | 1.0 |
| MTA1 | Metastasis associated 1 | 2.4 | ± | 1.2 |  | 2.9 | ± | 1.2 |  | 2.0 | ± | 1.5 |
| MTA2 | Metastasis associated 1 family, member 2 | 2.4 | ± | 1.8 |  | 2.4 | ± | 1.0 |  | 1.1 | ± | 0.8 |
| MTSS1 | Metastasis suppressor 1 | 2.6 | ± | 1.5 |  | 2.2 | ± | 1.3 |  | 2.6 | ± | 2.4 |
| MYC | V-myc myelocytomatosis viral oncogene homolog (avian) | 7.3 | ± | 4.4 |  | 12.5 | ± | 10.8 |  | 3.4 | ± | 1.5 |
| NFKB1 | Nuclear factor of kappa light polypeptide gene enhancer in B-cells 1 | 1.7 | ± | 0.8 |  | 1.3 | ± | 0.5 |  | 1.5 | ± | 0.9 |
| NFKBIA | Nuclear factor of kappa light polypeptide gene enhancer in B-cells inhibitor, alpha | 1.3 | ± | 0.9 |  | 1.5 | ± | 1.2 |  | 1.1 | ± | 0.9 |
| NME1 | Non-metastatic cells 1, protein (NM23A) expressed in | 2.0 | ± | 1.2 |  | 2.8 | ± | 1.5 |  | 2.7 | ± | 2.5 |
| NME4 | Non-metastatic cells 4, protein expressed in | 2.9 | ± | 2.2 |  | 2.7 | ± | 1.2 |  | 2.4 | ± | 0.9 |
| PDGFA | Platelet-derived growth factor alpha polypeptide | 1.5 | ± | 1.1 |  | 1.1 | ± | 0.9 |  | 0.9 | ± | 0.6 |
| PDGFB | Platelet-derived growth factor beta polypeptide | 2.5 | ± | 2.0 |  | 2.4 | ± | 1.2 |  | 2.5 | ± | 1.4 |
| PIK3R1 | Phosphoinositide-3-kinase, regulatory subunit 1 (alpha) | 2.1 | ± | 1.2 |  | 1.9 | ± | 0.8 |  | 2.0 | ± | 1.5 |
| PLAU | Plasminogen activator, urokinase | 1.8 | ± | 1.6 |  | 3.3 | ± | 2.3 |  | 1.9 | ± | 1.6 |
| PLAUR | Plasminogen activator, urokinase receptor | 1.2 | ± | 0.9 |  | 1.2 | ± | 0.6 |  | 0.7 | ± | 0.4 |
| PNN | Pinin, desmosome associated protein | 2.5 | ± | 1.0 |  | 2.3 | ± | 0.8 |  | 2.2 | ± | 1.9 |
| RAF1 | V-raf-1 murine leukemia viral oncogene homolog 1 | 1.7 | ± | 0.6 |  | 1.4 | ± | 0.5 |  | 1.4 | ± | 1.1 |
| RB1 | Retinoblastoma 1 | 1.8 | ± | 1.0 |  | 1.6 | ± | 0.8 |  | 1.3 | ± | 0.6 |
| S100A4 | S100 calcium binding protein A4 | 2.4 | ± | 1.9 |  | 2.2 | ± | 0.8 |  | 2.6 | ± | 2.2 |
| SERPINB5 | Serpin peptidase inhibitor, clade B (ovalbumin), member 5 | 12.1 | ± | 8.8 |  | 2.3 | ± | 1.5 |  | 13.3 | ± | 11.2 |
| SERPINE1 | Serpin peptidase inhibitor, clade E (nexin, plasminogen activator inhibitor type 1), member 1 | 2.3 | ± | 1.7 |  | 6.3 | ± | 5.7 |  | 1.2 | ± | 0.8 |
| SNCG | Synuclein, gamma (breast cancer-specific protein 1) | 1.3 | ± | 0.8 |  | 2.2 | ± | 1.5 |  | 1.1 | ± | 0.6 |
| SYK | Spleen tyrosine kinase | 3.2 | ± | 2.9 |  | 1.9 | ± | 1.4 |  | 1.7 | ± | 1.2 |
| TEK | TEK tyrosine kinase, endothelial | 1.2 | ± | 0.6 |  | 1.4 | ± | 0.9 |  | 1.1 | ± | 0.6 |
| TGFB1 | Transforming growth factor, beta 1 | 1.1 | ± | 0.5 |  | 1.1 | ± | 0.4 |  | 0.9 | ± | 0.9 |
| TGFBR1 | Transforming growth factor, beta receptor 1 | 1.8 | ± | 0.7 |  | 1.9 | ± | 0.7 |  | 2.1 | ± | 1.7 |
| THBS1 | Thrombospondin 1 | 1.7 | ± | 0.9 |  | 1.5 | ± | 1.2 |  | 1.0 | ± | 0.5 |
| TIMP1 | TIMP metallopeptidase inhibitor 1 | 4.1 | ± | 1.5 |  | 3.8 | ± | 1.6 |  | 3.9 | ± | 2.4 |
| TNF | Tumor necrosis factor | 1.2 | ± | 0.6 |  | 1.8 | ± | 1.4 |  | 1.5 | ± | 1.2 |
| TNFRSF10B | Tumor necrosis factor receptor superfamily, member 10b | 3.4 | ± | 1.8 |  | 3.3 | ± | 1.8 |  | 2.9 | ± | 2.9 |
| TNFRSF1A | Tumor necrosis factor receptor superfamily, member 1A | 0.9 | ± | 0.3 |  | 0.9 | ± | 0.5 |  | 0.9 | ± | 0.6 |
| TNFRSF25 | Tumor necrosis factor receptor superfamily, member 25 | 2.6 | ± | 1.7 |  | 2.6 | ± | 2.0 |  | 1.0 | ± | 1.0 |
| TP53 | Tumor protein p53 | 3.3 | ± | 1.8 |  | 2.6 | ± | 1.6 |  | 2.5 | ± | 1.3 |
| EPDR1 | Ependymin related protein 1 (zebrafish) | 3.6 | ± | 2.7 |  | 3.1 | ± | 2.1 |  | 3.8 | ± | 3.1 |
| VEGFA | Vascular endothelial growth factor A | 2.2 | ± | 1.9 |  | 2.4 | ± | 1.5 |  | 1.2 | ± | 0.7 |
| ADAMTS1 | ADAM metallopeptidase with thrombospondin type 1 motif, 1 | 1.2 | ± | 0.9 |  | 3.5 | ± | 2.8 |  | 1.0 | ± | 0.8 |
| CD44 | CD44 molecule (Indian blood group) | 6.9 | ± | 5.1 |  | 6.7 | ± | 3.2 |  | 9.2 | ± | 5.8 |
| CDH1 | Cadherin 1, type 1, E-cadherin (epithelial) | 0.9 | ± | 0.4 |  | 1.3 | ± | 1.0 |  | 1.1 | ± | 0.6 |
| COL12A1 | Collagen, type XII, alpha 1 | 2.4 | ± | 1.9 |  | 13.3 | ± | 11.9 |  | 6.7 | ± | 5.4 |
| COL14A1 | Collagen, type XIV, alpha 1 | 1.0 | ± | 1.0 |  | 2.4 | ± | 2.2 |  | 0.7 | ± | 0.6 |
| COL15A1 | Collagen, type XV, alpha 1 | 1.1 | ± | 0.6 |  | 2.1 | ± | 1.7 |  | 1.3 | ± | 0.6 |
| COL16A1 | Collagen, type XVI, alpha 1 | 1.3 | ± | 0.6 |  | 2.3 | ± | 1.1 |  | 1.7 | ± | 1.1 |
| COL1A1 | Collagen, type I, alpha 1 | 1.5 | ± | 0.9 |  | 5.0 | ± | 4.1 |  | 2.5 | ± | 1.9 |
| COL4A2 | Collagen, type IV, alpha 2 | 1.2 | ± | 0.6 |  | 3.2 | ± | 2.8 |  | 1.7 | ± | 1.2 |
| COL5A1 | Collagen, type V, alpha 1 | 1.1 | ± | 0.6 |  | 3.1 | ± | 2.4 |  | 1.6 | ± | 1.0 |
| COL6A1 | Collagen, type VI, alpha 1 | 0.9 | ± | 0.4 |  | 2.2 | ± | 2.1 |  | 1.4 | ± | 1.3 |
| COL6A2 | Collagen, type VI, alpha 2 | 1.1 | ± | 0.8 |  | 2.3 | ± | 2.3 |  | 1.1 | ± | 0.6 |
| VCAN | Versican | 1.1 | ± | 0.8 |  | 1.6 | ± | 1.6 |  | 0.9 | ± | 0.7 |
| CTGF | Connective tissue growth factor | 1.5 | ± | 1.0 |  | 4.2 | ± | 3.5 |  | 1.0 | ± | 0.7 |
| CTNNA1 | Catenin (cadherin-associated protein), alpha 1, 102 kDa | 0.8 | ± | 0.3 |  | 1.1 | ± | 0.8 |  | 0.9 | ± | 0.5 |
| CTNNB1 | Catenin (cadherin-associated protein), beta 1, 88 kDa | 1.6 | ± | 1.1 |  | 2.4 | ± | 1.3 |  | 2.1 | ± | 2.0 |
| CTNND1 | Catenin (cadherin-associated protein), delta 1 | 1.6 | ± | 0.8 |  | 1.9 | ± | 1.1 |  | 1.4 | ± | 1.2 |
| ECM1 | Extracellular matrix protein 1 | 7.9 | ± | 5.4 |  | 6.1 | ± | 3.3 |  | 9.2 | ± | 4.6 |
| FN1 | Fibronectin 1 | 0.8 | ± | 0.5 |  | 2.6 | ± | 2.2 |  | 0.9 | ± | 0.8 |
| ICAM1 | Intercellular adhesion molecule 1 | 1.5 | ± | 1.2 |  | 3.8 | ± | 2.5 |  | 1.5 | ± | 0.8 |
| ITGA5 | Integrin, alpha 5 (fibronectin receptor, alpha polypeptide) | 0.9 | ± | 0.5 |  | 2.6 | ± | 2.1 |  | 0.9 | ± | 0.5 |
| ITGA6 | Integrin, alpha 6 | 1.7 | ± | 0.8 |  | 1.9 | ± | 0.9 |  | 2.6 | ± | 1.7 |
| ITGA8 | Integrin, alpha 8 | 1.0 | ± | 0.5 |  | 1.4 | ± | 0.9 |  | 0.8 | ± | 0.3 |
| ITGAL | Integrin, alpha L (antigen CD11A (p180), lymphocyte function-associated antigen 1; alpha polypeptide) | 1.5 | ± | 1.1 |  | 1.7 | ± | 1.2 |  | 0.7 | ± | 0.4 |
| ITGAM | Integrin, alpha M (complement component 3 receptor 3 subunit) | 0.9 | ± | 0.5 |  | 1.4 | ± | 0.9 |  | 0.9 | ± | 0.7 |
| ITGB2 | Integrin, beta 2 (complement component 3 receptor 3 and 4 subunit) | 0.8 | ± | 0.4 |  | 1.1 | ± | 0.8 |  | 0.9 | ± | 0.8 |
| ITGB4 | Integrin, beta 4 | 2.4 | ± | 1.5 |  | 3.7 | ± | 2.9 |  | 2.7 | ± | 2.1 |
| LAMA1 | Laminin, alpha 1 | 0.8 | ± | 0.6 |  | 0.2 | ± | 0.2 |  | 0.4 | ± | 0.3 |
| LAMA2 | Laminin, alpha 2 | 1.5 | ± | 1.1 |  | 1.4 | ± | 0.9 |  | 1.3 | ± | 1.1 |
| LAMA3 | Laminin, alpha 3 | 1.1 | ± | 0.5 |  | 0.8 | ± | 0.5 |  | 1.4 | ± | 0.6 |
| LAMB1 | Laminin, beta 1 | 3.3 | ± | 2.7 |  | 3.9 | ± | 2.6 |  | 3.5 | ± | 2.2 |
| LAMB3 | Laminin, beta 3 | 2.0 | ± | 1.1 |  | 2.5 | ± | 1.0 |  | 2.1 | ± | 1.2 |
| LAMC1 | Laminin, gamma 1 (formerly LAMB2) | 1.3 | ± | 0.8 |  | 1.9 | ± | 1.3 |  | 1.4 | ± | 0.9 |
| MMP10 | Matrix metallopeptidase 10 (stromelysin 2) | 1.4 | ± | 0.8 |  | 3.4 | ± | 2.9 |  | 10.2 | ± | 9.6 |
| MMP11 | Matrix metallopeptidase 11 (stromelysin 3) | 5.2 | ± | 4.0 |  | 7.7 | ± | 6.4 |  | 4.7 | ± | 3.7 |
| MMP12 | Matrix metallopeptidase 12 (macrophage elastase) | 0.9 | ± | 0.4 |  | 3.2 | ± | 2.2 |  | 1.7 | ± | 0.9 |
| MMP14 | Matrix metallopeptidase 14 (membrane-inserted) | 2.3 | ± | 1.9 |  | 4.6 | ± | 2.7 |  | 2.7 | ± | 2.5 |
| MMP3 | Matrix metallopeptidase 3 (stromelysin 1, progelatinase) | 0.7 | ± | 0.4 |  | 4.4 | ± | 3.0 |  | 9.2 | ± | 9.6 |
| NCAM1 | Neural cell adhesion molecule 1 | 1.0 | ± | 0.6 |  | 2.3 | ± | 2.0 |  | 1.0 | ± | 0.8 |
| PECAM1 | Platelet/endothelial cell adhesion molecule | 0.8 | ± | 0.7 |  | 2.5 | ± | 2.6 |  | 0.9 | ± | 0.6 |
| SELE | Selectin E | 1.5 | ± | 1.0 |  | 2.9 | ± | 2.7 |  | 1.2 | ± | 0.7 |
| SELL | Selectin L | 1.3 | ± | 1.1 |  | 3.0 | ± | 2.8 |  | 0.7 | ± | 0.5 |
| SELP | Selectin P (granule membrane protein 140 kDa, antigen CD62) | 0.9 | ± | 0.5 |  | 3.7 | ± | 3.1 |  | 0.6 | ± | 0.5 |
| SGCE | Sarcoglycan, epsilon | 0.6 | ± | 0.4 |  | 1.0 | ± | 0.7 |  | 0.5 | ± | 0.3 |
| SPARC | Secreted protein, acidic, cysteine-rich (osteonectin) | 1.6 | ± | 0.9 |  | 4.0 | ± | 3.4 |  | 2.0 | ± | 1.2 |
| SPG7 | Spastic paraplegia 7 (pure and complicated autosomal recessive) | 2.0 | ± | 1.3 |  | 3.1 | ± | 2.1 |  | 1.9 | ± | 1.1 |
| SPP1 | Secreted phosphoprotein 1 | 0.9 | ± | 0.8 |  | 3.6 | ± | 2.7 |  | 1.7 | ± | 1.6 |
| THBS2 | Thrombospondin 2 | 2.0 | ± | 1.4 |  | 4.4 | ± | 4.1 |  | 2.5 | ± | 2.1 |
| TIMP2 | TIMP metallopeptidase inhibitor 2 | 1.5 | ± | 1.0 |  | 1.8 | ± | 1.1 |  | 1.0 | ± | 0.8 |
| CLEC3B | C-type lectin domain family 3, member B | 1.1 | ± | 0.7 |  | 1.9 | ± | 1.1 |  | 1.0 | ± | 0.9 |
| TNC | Tenascin C | 1.8 | ± | 1.6 |  | 2.9 | ± | 2.3 |  | 2.5 | ± | 2.0 |
| VCAM1 | Vascular cell adhesion molecule 1 | 1.1 | ± | 0.5 |  | 1.4 | ± | 0.9 |  | 0.6 | ± | 0.4 |
|  |  |  |  |  |  |  |  |  |  |  |  |  |
